# Importance of replication in analyzing time-series gene expression data: Corticosteroid dynamics and circadian patterns in rat liver

**DOI:** 10.1186/1471-2105-11-279

**Published:** 2010-05-26

**Authors:** Tung T Nguyen, Richard R Almon, Debra C DuBois, William J Jusko, Ioannis P Androulakis

**Affiliations:** 1 BioMaPS Institute for Quantitative Biology, Rutgers University, Piscataway, New Jersey, USA; 2 Biomedical Engineering Department, Rutgers University, Piscataway, New Jersey, USA; 3 Chemical & Biochemical Engineering Department, Rutgers University, Piscataway, New Jersey, USA; 4 Department of Pharmaceutical Sciences, State University of New York at Buffalo, Buffalo, New York, USA; 5 Department of Biological Sciences, State University of New York at Buffalo, Buffalo, New York, USA; 6 New York State Center of Excellence in Bioinformatics and Life Sciences, Buffalo, New York, USA

## Abstract

**Background:**

Microarray technology is a powerful and widely accepted experimental technique in molecular biology that allows studying genome wide transcriptional responses. However, experimental data usually contain potential sources of uncertainty and thus many experiments are now designed with repeated measurements to better assess such inherent variability. Many computational methods have been proposed to account for the variability in replicates. As yet, there is no model to output expression profiles accounting for replicate information so that a variety of computational models that take the expression profiles as the input data can explore this information without any modification.

**Results:**

We propose a methodology which integrates replicate variability into expression profiles, to generate so-called 'true' expression profiles. The study addresses two issues: (i) develop a statistical model that can estimate 'true' expression profiles which are more robust than the average profile, and (ii) extend our previous micro-clustering which was designed specifically for clustering time-series expression data. The model utilizes a previously proposed error model and the concept of 'relative difference'. The clustering effectiveness is demonstrated through synthetic data where several methods are compared. We subsequently analyze *in vivo *rat data to elucidate circadian transcriptional dynamics as well as liver-specific corticosteroid induced changes in gene expression.

**Conclusions:**

We have proposed a model which integrates the error information from repeated measurements into the expression profiles. Through numerous synthetic and real time-series data, we demonstrated the ability of the approach to improve the clustering performance and assist in the identification and selection of informative expression motifs.

## Background

Global gene expression analysis using microarrays has become an essential tool to study genome-wide transcriptional responses. Although this high-throughput technology produces a huge volume of useful data, enabling researchers to study the response of thousands of genes simultaneously, it faces many potential sources of uncertainties (e.g. technical noise, experimental treatments, biological sampling) [1,2]. As such, a number of statistical methods have demonstrated that the information contained in replicates is a valuable asset in order to assign proper confidence levels [3-6]. Rocke et al. [7] proposed a model accounting for measurement error to model gene expression profiles which has been used often in conjunction with variance-stabilizing transformation [8-11] and model-based clustering [12,13]. Consequently, researchers are designing more experiments with repeated measurements per gene per chip even though it is significantly more costly and time consuming. However, properly incorporating the replicate information remains a challenge.

A typical step in analyzing gene microarray data involves filtering for differential expression [14]. A number of methods have been proposed in this direction demonstrating the extensive insight gained in utilizing the information from replicates for determining the change of gene expression values e.g. t-test [15-17], ANOVA [18,19], SAM [20], EDGE [21]. An equally important part of the analysis is clustering which has been proven to be a powerful tool to rationalize transcriptional responses, identify possible functional relationships among them, and further elucidate important transcription factors as well as relevant biological pathways [13]. However, most clustering methods do not take into account the variability of gene expression profiles in the form of replicates. Variability is usually lumped into a mean effect and expression profiles are clustered based on average values of independently repeated measurements for each gene, thus missing, potentially, useful information [12].

Given that replicates can provide important insights into the nature of inherent variability among gene expression profiles [3], recent approaches have attempted to incorporate repeated measurements. There are two primary ways to handle replicated data: (i) indirectly integrate the error information among replicates into a pairwise similarity metric between two expression profiles to produce a more robust distance metric, and (ii) directly integrate the replicate information into clustering models. The former offers a relative advantage since clustering methods that take the distance metric as input can be utilized without any modification e.g. standard deviation-weighted correlation coefficient [22], shrinkage correlation coefficient [23]. Meanwhile, various models have been proposed for (ii) including those whose design centers around a specific statistical model (e.g. Bayesian mixture model [24,25], linear mixed model [12], random-effects model [13]) and those that are more general (e.g. CORE [26], trajectory clustering [27], mass distributed clustering [28]). Although such approaches produce more promising results, they are limited in that only a small number of computational methods can explore this information while many others requiring expression profiles as the input cannot.

In the present study we address a somewhat different question, namely whether we can integrate the error information into the time-series expression profiles so that we can utilize a variety of computational models [29-31] that take the expression profiles as the required input without any modification while taking into account the advantage of using replicated data (especially for clustering methods e.g. mclust [32], som [33], micro-clustering [34], consensus clustering [35], etc.).

The most straightforward approach to estimate time-series gene expression profiles is by computing the average expression levels over all replicates for each gene at each time-point (or condition in general). Of course, this approach does not properly take into account the variability in repeated measurements [23,36]. Therefore, in an attempt to estimate more robust expression profiles that integrate the error information from replicates, so-called 'true' expression profiles, we explore the error model [22] to estimate the 'true' mean expression value of a gene across all time-points and the concept of 'relative difference' driven by the theory of t-statistic [16,20] to compute the difference between the 'true' mean expression value across all time-points and the mean expression value at each time-point. Those relative differences are then used to infer the 'true' expression profile of the gene. Alternatively, we also explore the capability of using spline to find 'smoothing' expression profiles that take into account all repeated measurements [37].

We next demonstrate the effects of using the 'true' expression profiles in conjunction with clustering algorithms through synthetic and real time-series expression data. Following the convention of previous studies [23,36], we generated synthetic microarray data with known structure of classes and used the adjusted Rand index [36,38] to evaluate the performance of clustering via three popular clustering methods: hierarchical clustering, partitional clustering (kmeans [39], pam [40]) and model-based clustering (mclust [32]). Finally, we extend our earlier work that proposed a micro-clustering approach designed specifically for clustering time-series expression data [34,41]. The approach involves two main steps: (1) a fine grained clustering step to identify an extensive list of putative clusters based on a symbolic transformation, and (2) a selection step aiming at the determination of which clusters are significant as representative of the underlying response. Additionally, we also propose a heuristic to automatically select the parameter values for the clustering method. For the fine grained clustering step, the basic formalism of **S**ymbolic **A**ggregate appro**X**imation of time-series (SAX) has been adopted and modified [42,43]. Each 'true' expression profile is transformed to a corresponding sequence of symbols and then hashed to a particular motif value. As a result, all expression profiles with similar expression patterns will have identical symbolic representations and thus will be assigned to the same cluster.

However, the fine grained clustering step produces a large number of putative clusters while many of them are not significant enough to be considered as a representative expression pattern. Therefore, we propose a selection step based on the hypothesis that significant expression patterns will more likely consist of a large number of individuals compared to random data, given a threshold (p-value). As a result, only those clusters with a large-enough (based on the corresponding p-value) sizes are reported for subsequent investigations. Furthermore, due to the symbolic transformation heuristic of SAX the approach may produce several clusters with similar expression patterns and thus we also provide a heuristic to merge such clusters based on a criterion of maximizing the total homogeneity and separation of selected clusters.

Our results on synthetic data demonstrate that the clustering performance using 'true' expression profiles is superior to that when using average expression profiles and also to other methods with integrated error information. The output of this process can be used as input to a variety of other clustering methods without any modification while taking into account the information content in replicated data. Finally, we derive 'true' profiles for three real (rat liver) time-series datasets (acute/chronic corticosteroid administration [30,44] and circadian [45]) and the explore the extended version of micro-clustering to select significant patterns of transcriptional response. Computational results are further validated predicated upon literature evidence.

## Methods

### The 'true' expression profiles

In order to utilize a variety of computational models that take the expression profiles as the required input without any modification while taking into account the information of repeated measurements, we will estimate a more robust expression profile that integrate the error information from replicates. Let us assume that the 'average' time-series expression profile of gene i across T time-points with R_t _replicates at each time-point can be generally represented as

The subscripts i, t, r indicate the gene id, time, and replicate respectively. The procedure to estimate the 'true' expression profile consists of two main steps:

#### i. Estimate the 'true' mean expression value of a gene across all time-points

Utilizing the variance (error) of repeated measurements at each time-point σ_it_, the error model weights the average expression values at each time-point when computing the mean expression value of the gene across all time-points [22](1)

The variance of  can be calculated in two ways: one is to propagate the errors σ_it _and the other is from the scatter of  around (2)

The propagation of variance σ_p _is based on the error estimation of each individual time-point, leading to bias and/or systematic uncertainties whereas the other σ_s _has large fluctuation when the number of measurements is small although it is an unbiased measure. Statistically one can combine these two variances in estimation of the variance for [22](3)

#### ii. Estimate the relative difference between the 'true' mean expression value across all time-points and that at each time-point (one is replaced for the 'true' mean expression value)

In order to infer the expression value at each time-point of a gene, we utilized the concept of 'relative difference' [16,20] from the t-statistic to estimate its difference from the 'true' mean expression value of the gene. Let d_it _represent the relative difference between the 'true' mean expression value across all time-points and the mean value at a specific time-point:(4)

where s_t _is the standard deviation of these two quantities(5)

And thus, we propose a more accurate estimation of the average expression value at a specific time-point as follows(6)

**Figure 1:**
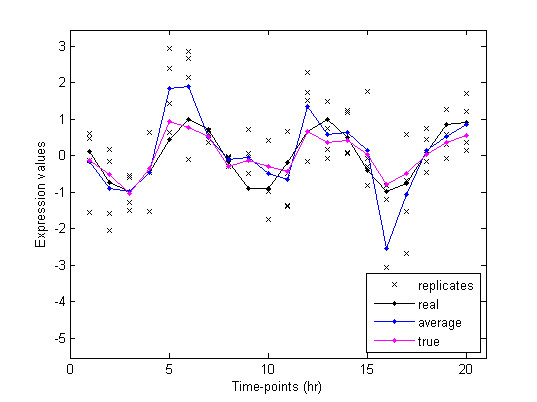
**The 'true' expression profiles are more robust than the average ones 'real' is the actual profile from simulated data without noise**. 'replicates' are obtained when noise is added to the actual value at each time-point. The average profile is showed to be more deviated from the actual profile than the 'true' profile.

As we rationalized the importance of microarray replicates in the background section, we hypothesize that the expression profiles would be more robust if there is some statistical approach that integrates the error information from replicates into the estimation. For average expression profiles, the expression value at a specific time-point is . In a similar manner we obtain formula (6) in a way that integrates the error information into two parts of the formula;  the part is the 'true' mean expression value across all time-points and the latter part d_it _is the relative difference between the 'true' mean expression across all time-points and the one at that specific time-point. Figure 1 compares the 'true' expression profile to the average one. Its effectiveness will be further demonstrated with the clustering performance on synthetic and real data.

### A combined computational framework of clustering and selection

#### i. Fine-grained clustering

**Figure 2:**
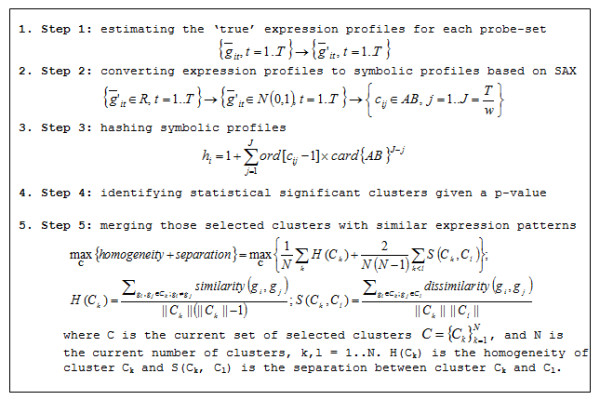
**Computational framework for clustering and selection**.

A very successful approach based on the **S**ymbolic **A**ggregation appro**X**imation - SAX [42] is applied to cluster time-series expression data. For SAX, temporal expression profiles are transformed into an appropriate sequence of symbols. Due to the nature of the discrete representation of the symbolic transformation, expression profiles are first z-score normalized to have a mean of 0 and a standard deviation of 1. Empirical testing [46] showed that such transformed subsequences have highly Gaussian distribution, and thus an equiprobable discretization technique is then applied to the vertical axis representing the expression values to obtain a number of intervals of expression values. The breakpoints are defined so that the area of regions defined by these breakpoints under the Gaussian curve are equal [42]. For example, to break the area under the Gaussian curve N(0,1) into three equal-area regions, the breakpoints would be -0.43 and 0.43. Each interval between two breakpoints is now assigned with a symbol, i.e. a character that belongs to a pre-defined alphabet set {AB}, and the expression value at each time-point t of a gene is replaced by a corresponding symbol. If so desired, the dimensionality of the data can be reduced through a 'piecewise aggregation approximation' [42] with a word-size w to reduce the temporal dimension from T to J = T/w. As a result, every normalized expression profile is approximated to a finite sequence of symbols *g*_*i *_= {*c*_*ij *_∈ *AB*, *j *= 1.. *J*} (Figure 2, step 2) [41].

Once represented by a sequence of symbols, each expression profile is 'hashed' to an integer motif value which is also an identifier corresponding to a cluster  (ord[] is the order of the character c_ij _in the alphabet set AB and card{} returns the number of elements in set AB). The only difference between our hashing formula and the original one is the change in weighting significant signals. We weight the differences of the sequence at the beginning heavier than that of the end. Consistent with this is the observation that the signals that are correlated at early time-points will be more closely related than those that are correlated at the end of the time series [34]. As a result, we attain a number of clusters for the data which is up to two most important parameters of the hashing formula i.e. the alphabet size α = card{AB} and the word size w (Figure 2, step 3) (a more detail discussion is presented in [41]).

#### ii. Selection of significant clusters

The fine-grained clustering step assigns a unique identifier to all transcriptional profiles so that genes with similar expression profiles will be hashed to the same motif values. However, the approach generates a large number of identifiers due to the nature of the hash formula, resulting in a huge number of clusters with many trivial ones i.e. clusters with sizes too small to be considered as significant expression patterns of transcriptional responses. Therefore, following our previous work [35] we assign each cluster a simple hypothetical quantity called 'cluster significance' which is simply the cluster size. In order to select significant clusters, we first estimate the distribution of cluster significance using random data and then compute the p-value for each cluster above. The data after step 1 is randomly resampled (based on the convex-hull approach [47]), and the entire process starting from step 2 to step 3 is run with the same parameters. The process is repeated nr times (nr = 100 in this study) and we get totally N random clusters. Because of the underlying equiprobable distribution associated with SAX, random expression profiles will be assigned to different hash values with equal probability, resulting in the small size for almost all random clusters. Therefore, we hypothesized that the smaller the size of the cluster, the more likely the corresponding clusters are to be random. Based on this hypothesis, the p-value of a cluster with size 's' is defined as the total number of random clusters with the sizes at least s over N random clusters. As a result, given a p-value we can infer the cluster-size cutoff for the selection process and only those clusters whose sizes are larger than that cutoff are reported as significant clusters for further investigation (Figure 2, step 4).

#### iii. Merging similar expression patterns

Because converting an expression profile to a sequence of symbols is an approximation, related expression profiles may be hashed to similar (not the same) sequences of symbols albeit different motif values. Consequently, resulting clusters can have similar expression patterns but assigned to two or more clusters. Since the cluster homogeneity reflects how similar are expression profiles in the same cluster whereas cluster separation quantifies how well different expression profiles are separated, we propose an optional procedure in order to merge similar clusters together based on the assumption that the sum of homogeneity and separation of all final clusters is maximized (Figure 2, step 5). Starting with all significantly selected clusters, the procedure searches for a grouping of two clusters so that their combination can generate a maximal increase of the sum of homogeneity and separation of all current clusters after merging those two clusters. The process is repeated until no more combinations are found i.e. any new combination always reduces the sum of homogeneity and separation. Eventually, a list of significant expression patterns that characterize the underlying transcriptional response is generated.

## Materials

### Synthetic data

Following the convention of previous studies [23,36], we generate synthetic data which contain 6 clusters of genes, each of which consists of 66 genes across T = 20 time-points. Four of six clusters are generated using the sine function plus some noise

and the other two are generated following a non-periodic linear function plus some noise

Here the subscript m denotes the cluster number and i, t, r indicate the gene id, the time, and the replicate numbers respectively. Therefore, {g_itr_} is a synthetic expression profile of a simulated gene with r replicates for each of T time-points. The parameters ω_m _and φ_m _represent the random wavelength and random shift for cluster m (*ω*_*m *_∈ [0.5*π*, 5*π*], *φ*_*m *_∈ [0,2*π*]). α is the level of noise which is 1.0 for low noise and 2.5 for high noise in this study. The parameters σ_i _and σ_it _represent the error levels for gene i and for experiment at time-point t which are randomly drawn from a uniform distribution in the interval [0.2, 1.2]. Finally, x_itr _is a random variable drawn from a standard normal distribution to create the variability for replicates.

### Acute corticosteroid data

Forty-seven male ADX Wistar rats weighting from 225 to 250 g underwent right jugular vein cannulation under light ether anesthesia 1 day before the study [30]. Forty-three rats were injected with a single intravenous bolus dose of methylprednisolone (MPL) of 50 mg/kg. Animals were sacrificed by exsanguinations under anesthesia and liver samples were harvested at 0.25, 0.5, 0.75, 1, 2, 4, 5, 5.5, 6, 7, 8, 12, 18, 30, 48, and 72 after dosing. The sampling time points were selected based on preliminary studies describing GR dynamics and enzyme induction in liver. Four untreated rats were randomly sacrificed as controls. The gene expression was obtained via the Affymetrix RG-U34A array which consists of 8,799 probesets. The data are publicly available through the GEO Omnibus Database (http://www.ncbi.nlm.nih.gov/geo/) under the accession number GDS253. After filtering by ANOVA (p-value = 0.05) [19,35], 2,920 probesets considered as differential expression are used for further analysis.

### Chronic corticosteroid data

In a similar experiment model, forty rats were administered a low level of 0.3 mg/kg/hr infusions of MPL over 168 h via an Azlet pump [44]. The pump drug solutions were prepared for each rat based on its predose body weight. Animals were sacrificed at various times up to 7 days; specifically the time-points included are 6, 10, 13, 18, 24, 36, 48, 72, 96, and 168 h. A control group of four animals was implanted with a saline-filled pump and killed at various times throughout the 7-day study period. Unlike the previous experiment, the microarray platform for this dataset is the RAE230A which consists of 15,923 probesets. The data are publicly available through the GEO Omnibus Database under the accession number GDS972. After filtering by ANOVA (p-value = 0.05), 4,361 probesets are selected as significantly differentially expressed probesets for further analysis.

### Circadian data

To examine the fluctuations of gene expression patterns in liver within the 24 hour circadian cycle in normal animals, fifty four normal male Wistar rats (body weights ~ 225-275 g) were housed and allowed to acclimatize in a constant-temperature environments (22°C) equipped with 12 h light/dark cycle [45]. Twenty-seven rats (Group I) were acclimatized for 2 weeks prior to study to a normal light/dark cycle where lights went on at 8 AM and off at 8 PM whereas the other 27 rats (Group II) were acclimatized a reserved light/dark cycle where lights went on at 8 PM and off at 8 AM. Rats in Group I were killed in three successive days at 0.25, 1, 2, 4, 6, 8, 10, 11, 11.75 hr after lights on to capture the light period. Rats in Group II were killed on three successive days at 12.25, 13, 14, 16, 18, 20, 22, 23, 23.75 h after lights on to capture the dark period. Animals sacrificed at the same time on successive days were treated as triplicate measurements. The gene expression was obtained via the Affymetrix RAE230A array which consists of 15,923 probesets. The data are publicly available through the GEO Omnibus Database under the accession number GSE8988. After filtering by ANOVA (p-value = 0.05), 2,468 probesets considered as differential expression are used for further analysis.

## Results and Discussion

### The 'true' expression profile improves cluster quality on synthetic data

To evaluate the effectiveness of the 'true' expression profile compared to using the 'average' profile, we use the synthetic data with known class structure as described earlier. As in previous studies [23], we also assess the effect of the number of replicates on cluster quality. Each synthetic data contains 20 time-points with r replicates (r = 2, 3, 4, 5, 6, 7, 8, 9, 10) at each time-point and two different levels of noise (low and high). In addition to comparing the clustering performance using the 'true' profiles with the average profile, we also compare with several other methods that take into account error information from replicated data. Specifically, we measure cluster quality when using two typical similarity distance metrics which include the error information, namely the standard deviation (SD)-weighted correlation coefficient [22] and the shrinkage correlation coefficient [23]. Since our model generates expression profiles which are applicable to any clustering method, we also tested an alternative method which uses cubic splines to infer expression profiles which account for repeated measurements, so-called 'smoothing' profiles. For each gene, we establish two vectors - one consist of all replicates and another contains corresponding time-points. They are then input into function 'smooth.spline' in stats R package [37]; other parameters (e.g. the degree of freedom, smoothing parameters) are optimized from an internal 'generalized' cross-validation process provided by the tool. After that, the expression value at each time-point is inferred to create the 'smoothing' profile for the gene. Subsequently, the Pearson correlation coefficient is applied to estimate the similarity distance between two genes with the average profiles, the 'true' ones, and the 'smoothing' ones. After obtaining the pairwise distance matrix, we apply three popular clustering methods: hierarchical clustering (with average linkage option, available in MATLAB), partitional clustering (k-means [39], pam [40]), and model-based clustering (mclust [32]) to cluster the data into six clusters. In order to assess the clustering performance, we use the adjusted Rand index [36,38] which is a statistic that measures the extent of concurrence between the clustering results and the underlying known class structure. The larger the Rand index is, the higher the agreement between clustering results and prior knowledge of class structure i.e. better clustering performance.

**Figure 3:**
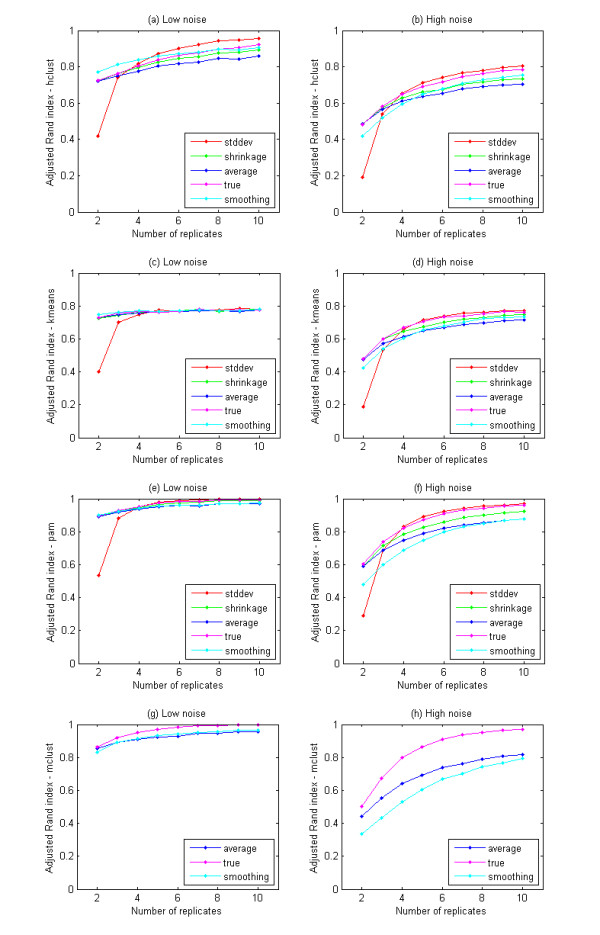
**The performance of typical clustering methods on different error-measurement integrated approaches**. 'stddev' represents for the clustering performance on synthetic data using the approach with the SD-weighted correlation coefficient metric; similarly, 'shrinkage' is for the approach with the shrinkage correlation coefficient metric; 'average' is for the clustering performance on average profiles; 'true' is for that on 'true' profiles; and 'smoothing' is for that when using method 'spline' to infer the expression profiles and then clustering. The horizontal axis shows the corresponding number of replicates in the dataset while the vertical axis demonstrates the clustering performance of the corresponding approach (the higher the better). Results are the average of clustering accuracies over 1000 randomly generated synthetic datasets.

Figure 3 depicts the clustering performance when using our proposed model compared to other approaches. We evaluate the average of 1000 randomly generated synthetic data sets. Figure 3a and 3b show the comparisons using hierarchical clustering. For the low-noise level (Figure 3a), the clustering performance using the 'true' profiles is slightly worse than that when using the SD-weighted correlation coefficient metric or 'smoothing' profiles. However, it is still much better than that when using the average profiles. For the high-noise level, it is comparable to the best achievable by any other method (Figure 3b). When other clustering methods are used (e.g. kmeans - Figure 3c &3d, pam - Figures 3e &3f, mclust - Figures 3g &3h), the clustering performance on the 'true' expression profiles is always superior, or comparable, to any other approach on both low and high noise data, and far better than that of the average profiles in high noise data. Additionally, when datasets are sampled with few time instants, the proposed approach is more advantageous than the alternative method that uses spline to infer expression profiles due to the 'overfitting' issue (detailed results in Additional File 1 and Additional File 2).

### Liver response to acute corticosteroid administration

**Figure 4:**
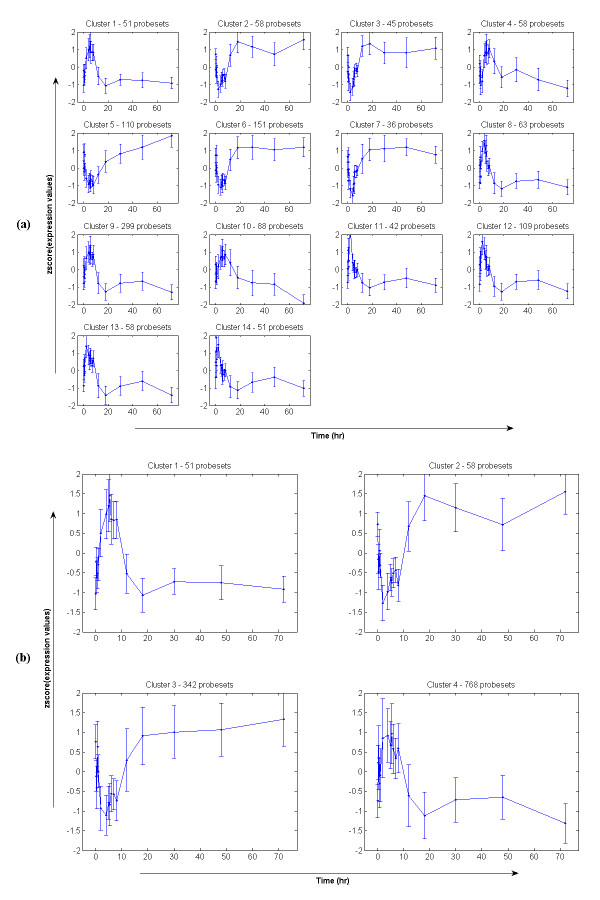
**Selected expression patterns from the acute corticosteroid dataset; (a) before merging and (b) after merging**. The horizontal axis is seventeen time-points (0, 0.25, 0.5, 0.75, 1, 2, 4, 5, 5.5, 6, 7, 8, 12, 18, 30, 48, 72 hours) and the vertical axis is the normalized (z-score) of expression values from 'true' expression profiles. Error bars are two standard deviations of expression values at each particular time-point.

We analyze 2,920 probesets that are significantly differentially expressed. Cluster analysis and selection with our framework (Figure 2) yields fourteen significant clusters given a p-value = 0.05 for the selection, corresponding to the cutoff cluster-size 34 (Figure 4a). The results presented here are based on the alphabet size α = 3 and the word size w = 3 (see **Statistical and Computational Issues **section). In total, we identify 1,219 probesets which are divided into two main expression patterns: early up or down regulation followed by returning to the baseline state. This is shown more clearly with the 4 clusters after merging (Figure 4b). In brief, clusters 1 & 4 (51 and 768 probesets respectively) exhibit an up-expression pattern. These clusters show an induction with a maximum at around 5 h with some fluctuation around the peak and then exhibit a fast decline to the baseline after about 18 h. Cluster 2 & 3 which consist of 58 and 342 probesets respectively exhibit a down-expression pattern. As depicted in Figure 4b, they exhibit a down-regulation during the first 5 h and then return to baseline at around 18 h. Generally, the progression of the transcriptional responses of the acute corticosteroid dataset is comprised of a deviation away from the baseline as the drug is injected into the system and an eventual return back to the baseline. This overall systemic response is similar to the response described by an indirect effect model presented in [48]. Although the drug is cleared within about 6 hours the longer time to return to baseline is due to a continuing cascade of events that were initiated by the drug but continue long after the drug is gone.

#### Liver response to chronic corticosteroid administration

**Figure 5:**
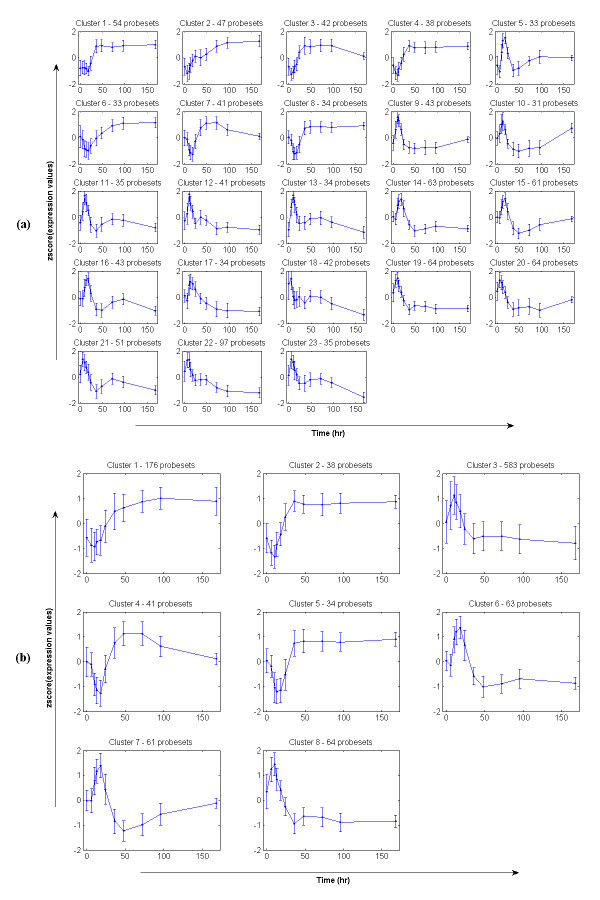
**Selected expression patterns from the chronic corticosteroid dataset; (a) before merging and (b) after merging**. The horizontal axis is eleven time-points (0, 6, 10, 13, 18, 24, 36, 48, 72, 96, 168 hours) and the vertical axis is the normalized (z-score) of expression values from 'true' expression profiles. Error bars are two standard deviations of expression values at each particular time-point.

Under a chronic administration of corticosteroids, 4,361 probesets are selected as differential expression using ANOVA (p-value = 0.05). Similarly, we apply the proposed model to convert these expression profiles to 'true' expression profiles and then further analyze them through our clustering and selection process. In this case, we also use the threshold p-value = 0.05 for the selection of significant clusters but the clustering parameters are different, specifically the alphabet size α = 3 and the word size w = 2 (see **Statistical and Computational Issues **section). Results are shown in Figure 5 with 23 clusters and a total number of 1,060 selected probesets (Figure 5a). After merging using the criterion of maximal sum of the homogeneity and separation, we obtain 8 clusters which show more clearly the patterns of the transcriptional responses when the drug is chronically administrated over a long period (Figure 5b). Generally, there are four main expression patterns which are very different from the transcriptional responses of acute corticosteroid administration. In brief, cluster 1, 2, and 5 which contain 176, 38, 34 probesets respectively characterize a pattern with a slightly early down-regulation early followed by a sustained up-regulation and eventual convergence to a new steady state in the presence of the drug. The second pattern characterized by cluster 3, 6, and 8 (583, 63, 64 probesets respectively) exhibits an induction of about 10 h and then down-regulated and stabilization to a new steady state. Cluster 4 (41 probesets) shows the third expression pattern which exhibits a simple repression with a maximum at around 18 h followed by an induction at around 50 h and a slower return and evolving dynamics as late as 168 h. Opposite with this, cluster 7 (61 probesets) shows the pattern that consists of a simple induction with a maximum at around 18 h followed by a repression at around 50 h and a similarly evolving dynamics.

### Circadian patterns in liver

**Figure 6:**
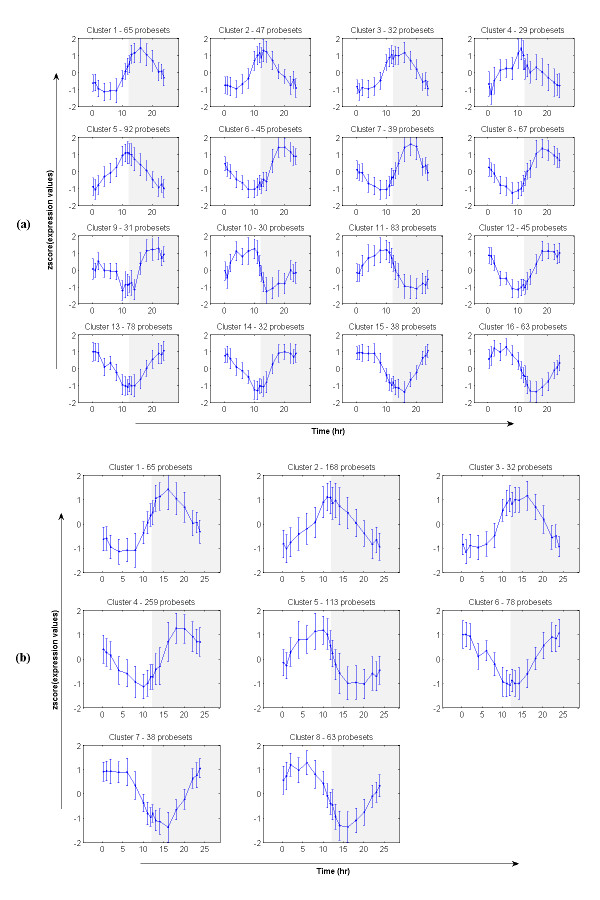
**Selected expression patterns from the circadian dataset; (a) before merging and (b) after merging**. The horizontal axis is eighteen time-points (0.25, 1, 2, 4, 6, 8, 10, 11, 11.75, 12.25, 13, 14, 16, 18, 20, 22, 23, 23.75 hours) and the vertical axis is the normalized (z-score) of expression values from 'true' expression profiles. Error bars are two standard deviations of expression values at each particular time-point.

In order to have a comparison between circadian regulated gene expression patterns with those directly regulated by corticosteroids, we provide here an analysis of circadian rhythms of mRNA expression in the liver of adult male rats. The dataset consists of 2,468 significantly differentially expressed probesets (filtered by ANOVA with p-value = 0.05) for which we further identify the 'true' expression profiles. Subsequently, we apply the proposed framework to cluster and select significant transcriptional responses with the alphabet size α = 3, the word size w = 3 (see **Statistical and Computational Issues **section), and the threshold p-value = 0.05 for the selection of significant clusters. We identify 816 probesets which are divided into 24 statistically significant expression patterns (Figure 6a). However, after the merging process we obtain eight main expression patterns (Figure 6b). In brief, cluster 1 (65 probesets) shows an early down-regulation at around 5 h (in the light period) and then up-regulation with a maximal peak at around 15 h (in the dark period). Similarly to this pattern, cluster 4 (259 probesets) shows a late down regulation at around 10 h and then up-regulation at around 20 h. In contrast to these two patterns, cluster 8 (63 probesets) and cluster 5 (113 probesets) present a pattern with early, and late respectively, up-regulation and then down regulation. Cluster 2 (168 probesets) and cluster 3 (32 probesets) are characterized by an simple induction with a maximum at around 12 h and 15 h respectively followed by a return to the baseline at 24 h. In the opposite direction, cluster 6 (78 probesets) and cluster 7 (38 probesets) show a simple repression with a maximum at around 10 h and 15 respectively. Therefore, without any assumption about the periodicity of the data it is still possible to capture the underlying transcriptional responses, i.e., expression patterns, within the data. Selected patterns are in concurrence with those in a previous report that assumes the periodicity [45].

### Statistical and computational issues

With the importance of information implicitly included in the replicates, several pairwise similarity distance metrics as well as particular clustering models have been proposed to take this information into account. However, unless these specific metrics are employed, replicate information cannot be utilized in conjunction with alternative methods. As an example, distance metrics that take replicates into account (e.g. stddev, shrinkage) are not applicable to model-based clustering (mclust), micro-clustering or any consensus-clustering method since these require the application of a collection of multiple, alternative, clustering methods. Therefore, we proposed a model to generate more robust gene expression profiles for general computational analyses so that they can be applied without any modification while still taking into account the replicate information. Alternatively, ones can explore the benefits from 'smoothing' expression profiles which are also expression profiles with integrated error information. However when 'smoothing' profiles are used in conjunction with clustering it is very critical to identify and select appropriate smoothing parameters. Due to the distribution of replicates around the mean expression values, 'smoothing' approaches can easily fail in detecting proper parameters to recover the actual profiles. As a result, the cluster quality using 'smoothing' profiles gets progressively worse on data with high-noise levels although it offers some advantages at low-noise levels.

The 'true' expression profile approach does not only consider the error information from repeated measurements at each time-point but also takes into account the dynamics of expression across all time-points when estimating the 'true' mean expression value of a gene . Such characteristics are best demonstrated through the examination of the clustering performance on the synthetic data. Generally, in all cases the clustering performance using the 'true' expression profiles is superior to that when the average profiles are used, suggesting that our proposed model which integrates the error information from repeated measurements into expression profiles offers clear advantage when used in clustering.

Regarding the micro-clustering, there are two most important parameters in our symbolic transformation step: the alphabet size 'α' and the word size 'w'. Different values of these parameters can lead to different clustering results. Therefore, we have proposed a heuristic to select the values for those parameters by defining a quantity, so-called 'the quality of the selection', that takes into account both the number of selected probesets and their coherence in selected clusters as follows

**Figure 7:**
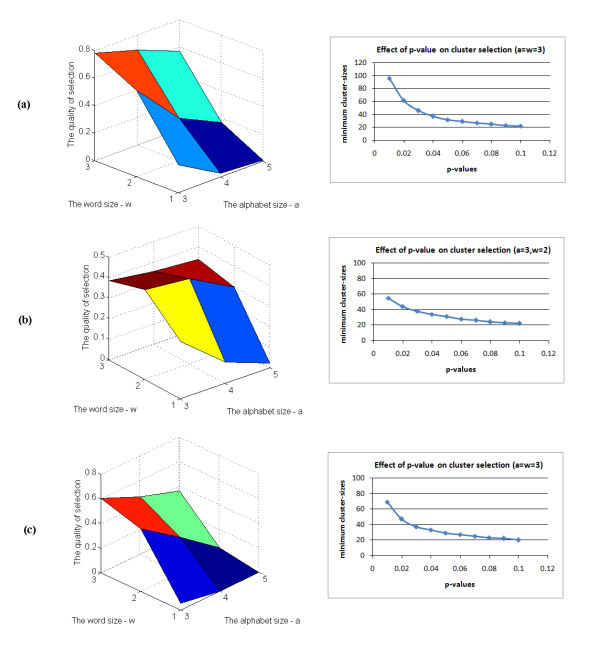
**Effects of parameters on the selection**. (a) Illustration results from the acute corticosteroid dataset (left is the optimal parameters for a given dataset and right is the corresponding cluster-size for a given p-value); (b) Illustration results from the chronic corticosteroid dataset; and (c) Illustration results from the circadian dataset.

The homogeneity and separation is estimated as in step 5 of the framework (Figure 2). For each dataset, we make an exhaustive search for all commonly used values of these two parameters ('α' from 3 to 5, 'w' from 1 to 3) and select the one corresponding to the maximal QS (Figure 7). The heuristic is applied in order to provide parameters for the clustering analysis of real time-series datasets used in this study. Besides, another important threshold is the significant p-value for cluster selection which can be inferred to corresponding cluster-size cutoff values (Figure 7). In this study, we considered only one value (p-value = 0.05) for this parameter in the selection process. As a result, given a dataset the proposed framework can automatically select the required parameters and do the analysis without any prior knowledge.

## Conclusions

We have proposed a statistical model that accounts for the variability in repeated measurements to estimate more robust expression profiles, so-called 'true' expression profiles. The effectiveness of the model has been demonstrated on synthetic data as the method that achieves superior and/or comparable clustering performance to that of other related approaches, especially much better to that when using the average expression profiles. The output of this representation can be used as a powerful input to a variety of computational models that require gene expression profiles as their input, especially when used in conjunction with clustering. Furthermore, we extend our prior micro-clustering algorithm, designed specifically for clustering time-series expression data, by developing a criterion for the selection of significant clusters; the merging of similar expression patterns; and providing a heuristic to identify parameters for optimal cluster selection. Results on real time-series gene expression data have demonstrated the effectiveness and usefulness of the approach.

## Authors' contributions

TTN designed the algorithms and experiments, devised and implemented the algorithms. RRA, DCD and WJJ reviewed the material and contributed to the discussion. IPA organized the activities and structured the approach. All authors all authors read and approved the final manuscript.

## Supplementary Material

Additional file 1 **Supplemental Data**. Provide detailed clustering results in this study, including cluster_id, probeset_id, gene_id and corresponding 'true' expression profiles of identified probesets in responses to acute/chronic corticosteroid administration and in circadian patterns. Detailed results of Figure 3 are also included. Click here for file

Additional file 2 **Expression Paterns**. Provide detailed clustering results in this study, including cluster_id, probeset_id, gene_id and corresponding 'true' expression profiles of identified probesets in responses to acute/chronic corticosteroid administration and in circadian patterns. Click here for file
